# Different Contribution of Monocyte- and Platelet-Derived Microvesicles to Endothelial Behavior

**DOI:** 10.3390/ijms23094811

**Published:** 2022-04-27

**Authors:** Marta Brambilla, Maria Talmon, Paola Canzano, Luigia G. Fresu, Sandra Brunelleschi, Elena Tremoli, Marina Camera

**Affiliations:** 1 Centro Cardiologico Monzino, Istituto di Ricerca e Cura a Carattere Scientifico, 20138 Milan, Italy; mbrambilla@ccfm.it (M.B.); pcanzano@ccfm.it (P.C.); 2 Department of Health Sciences, School of Medicine, University of Piemonte Orientale, 28100 Novara, Italy; maria.talmon@med.uniupo.it (M.T.); luigia.fresu@med.uniupo.it (L.G.F.); sandra.brunelleschi@med.uniupo.it (S.B.); 3 Maria Cecilia Hospital, 48033 Cotignola, Italy; etremoli@gvmnet.it; 4 Department of Pharmaceutical Sciences, Università Degli Studi di Milano, 20133 Milan, Italy

**Keywords:** microvesicles, endothelial cells, monocytes, platelets, coronary artery disease

## Abstract

Several contributions of circulating microvesicles (MVs) to the endothelial dysfunction have been reported in the past; a head-to-head comparison of platelet- and monocyte–derived MVs has however never been performed. To this aim, we assessed the involvement of these MVs in vessel damage related processes, i.e., oxidative stress, inflammation, and leukocyte-endothelial adhesion. Platelets and monocytes isolated from healthy subjects (HS, *n* = 15) were stimulated with TRAP-6 and LPS to release MVs that were added to human vascular endothelial cell (hECV) culture to evaluate superoxide anion production, inflammatory markers (IL-6, TNF*α*, NF-κB mRNA expression), and hECV adhesiveness. The effects of the MVs-induced from HS were compared to those induced by MVs spontaneously released from cells of patients with ST-segment elevation myocardial infarction (STEMI, *n* = 7). MVs released by HS-activated cells triggered a threefold increase in oxidative burst in a concentration-dependent manner. Only MVs released from monocytes doubled IL-6, TNF*α*, and NF-κB mRNA expression and monocyte-endothelial adhesion. Interestingly, the effects of the MVs isolated from STEMI-monocytes were not superimposable to previous ones except for adhesion to hECV. Conversely, MVs released from STEMI-platelets sustained both redox state and inflammatory phenotype. These data provide evidence that MVs released from activated and/or pathologic platelets and monocytes differently affect endothelial behavior, highlighting platelet-MVs as causative factors of impaired endothelial function in the acute phase of STEMI.

## 1. Introduction

Microvesicles (MVs) are 0.1–1 µM diameter extracellular particles that originate from almost all cell types constitutively or as result of a stimulus (e.g., inflammation, oxidative stress, hypoxia, aging, drug treatments) [1]. 

MVs act as mediators of cell-to-cell crosstalk [1] exerting different biological functions according to their cargo (e.g., lipid or protein mediators, mRNA, miRNAs, and short/long ncRNAs) that depend on their parent cells and on the stimuli that triggered their release [2]. 

MVs are key players in several pathological settings including inflammation, atherosclerosis, and thrombosis. Elevated levels of circulating MVs have been reported in patients with cardiovascular risk factors such as dyslipidemia [3], diabetes [4,5], hypertension [6], and smoking [7], as well as in those with cardiovascular events [8,9]. In addition, in patients with atherothrombosis and myocardial infarction [10,11], circulating MVs correlate with disease severity, and this highlights their potential role as predictive biomarkers for diagnosis and prognosis of diseases.

Studies carried out in patients with cardiovascular diseases have shown that MVs derived from platelets (Plt-MVs), which represent 70% to 90% of the circulating large extracellular vesicles, express high levels of membrane phosphatidylserine, P-selectin and tissue factor (TF) [12] and carry the procoagulant phenotype of activated platelets [13] thus sustaining processes such as thrombosis [14], inflammation [15], and angiogenesis [16]. Increased levels of MVs derived from platelets are associated with increased systemic oxidative stress, being able to decrease nitric oxide production [17]. Plt-MVs have also been shown to have a role in modulating cell interactions, as they increase the endothelial expression of Intercellular Adhesion Molecule-1 (ICAM-1) and monocyte chemotaxis by carrying arachidonic acid [18].

Although less investigated, monocytes-derived MVs (Mo-MVs) also contribute to the atherothrombotic event expressing high levels of TF and P-selectin glycoprotein ligand-1 [19]. They have critical role in inflammation [20,21] by stimulating monocytes themselves to produce inflammatory cytokines and oxygen reactive species [22], thus potentially activating platelets [19] and endothelial cells [23]. In particular, Mo-MVs transfer ICAM-1 to endothelial cells promoting leukocyte adhesion [24] and express high levels of IL-1β that in turn stimulate endothelial cells [25]. 

The contribution of circulating MVs to the endothelial dysfunction has been previously reported but a head-to-head comparison of platelet- and monocyte–derived MVs has never been performed. 

We explored the impact of MVs generated in vitro by platelets and monocytes from healthy subjects (HS), under resting conditions or upon activation—this latter condition was chosen in order to mimic endothelial perturbation—in terms of induction of oxidative stress, production of inflammatory cytokines, and upregulation of adhesive molecules.

In addition, the effect of MVs released by agonist stimulated cells was compared with that caused by MVs spontaneously released by cells isolated from patients with ST-elevation myocardial infarction (STEMI), a pathological condition characterized by a well-known inflammatory background [26,27].

## 2. Results

### 2.1. The In Vitro Ability to Generate MVs Is Higher in Platelets Than in Monocytes of Healthy Subjects

**Figure 1:**
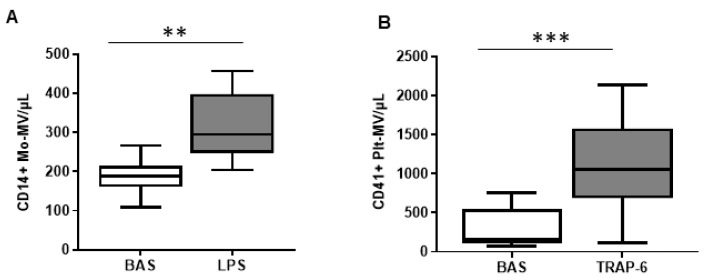
MVs released by monocytes and platelets isolated from healthy subjects. (**A**) Number of CD14^+^ MVs released by monocyte at resting conditions (basal) and after challenge with LPS 10 μg/mL (*n* = 10). (**B**) Number of CD41^+^ MVs released by platelets at resting conditions and after TRAP-6 20 μM challenge (*n* = 15). Data are represented as box and whisker plot (min to max) and analyzed by Mann–Whitney U test.** *p* < 0.01, *** *p* < 0.001 vs. resting condition.

The amount of MVs released by monocytes and platelets isolated from healthy subjects was assessed by flow cytometry. Stimulation of monocytes with LPS induced a two-fold increase in MV production compared to untreated cells (Figure 1A). Platelets released 6.7-fold more MVs after stimulation with TRAP-6 than at baseline (Figure 1B). Of note, the amount of MVs produced by unstimulated platelets (309 ± 250 MVs/μL) was almost double compared to that released by a physiologically comparable number of unstimulated monocytes (189 ± 43 CD14^+^ MVs/μL), even though platelets were left to release MVs for shorter periods of time. 

Overall, the in vitro production of MVs by monocytes and platelets, despite the different experimental conditions required by the two cell types, ultimately reflects the amount of vesicles found in vivo [11,28].

### 2.2. MVs Released by Activated Monocytes and Platelets Induce Oxidative Stress in Endothelial Cells

**Figure 2:**
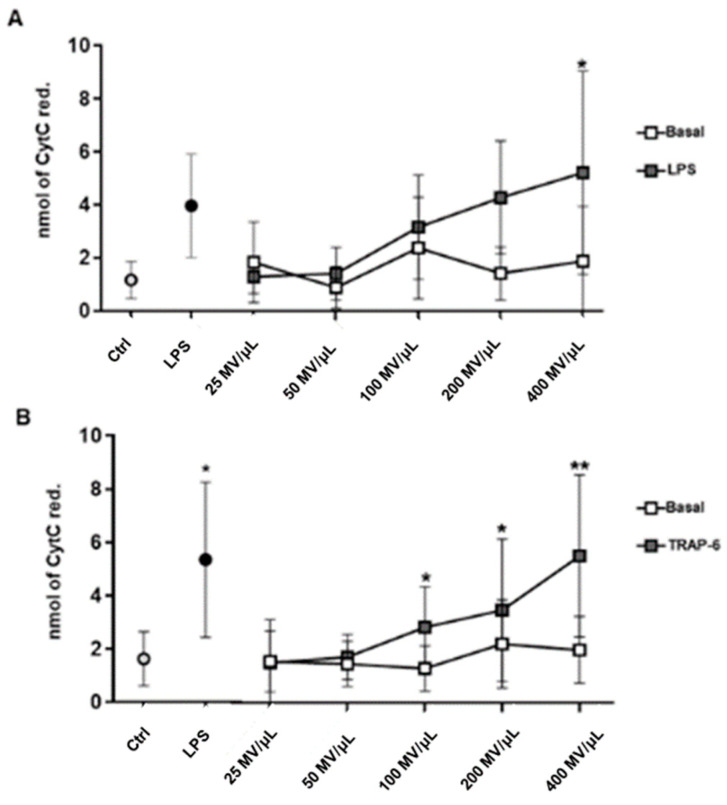
Monocyte- (Mo-) and platelet- (Plt-) MVs effect on superoxide anion production by hECV. Endothelial cells were incubated for 30 min with the reported concentration of Mo-MVs (**A**) and Plt-MVs (**B**) isolated from healthy subjects under resting conditions (basal) and upon stimulation with LPS (10 μg/mL) or TRAP-6 (20 μM). Data are expressed as nmoles of cytochrome C reduced/10^6^ cells/30 min and are represented as mean ± SD of 9 independent experiments and analyzed by one-way ANOVA with Dunnett’s test for multiple comparison. * *p* < 0.05; ** *p* < 0.01 vs. respective untreated cells (Ctrl).

Superoxide anion production, as marker of oxidative stress, was assessed in endothelial cells challenged with the Mo- and Plt-MVs released by both resting and activated cells. Data indicate that O_2_- production by human vascular endothelial cells (hECV) was not affected by Mo-MVs and Ptl-MVs produced by resting cells (from 25 to 400 MVs/µL) even at the highest concentration tested (Figure 2A,B). Conversely, LPS-induced Mo-MVs were able to induce superoxide anion production to levels comparable to those triggered by LPS treatment, used as a positive control (Figure 2A). Similarly, TRAP-6-induced Plt-MVs were effective in inducing oxidative stress being able to significantly modulate superoxide anion production by hECV in a concentration-dependent manner (Figure 2B).

These data indicate that the interaction of physiologically produced monocyte- and platelet-MVs with endothelium does not change its redox balance. In contrast, when MV release is induced by a surrounding pro-inflammatory milieu, MVs, produced by either cell types, acquire a comparable pro-oxidative activity.

### 2.3. Monocyte-MVs More Than Platelet-MVs Induce the Expression of Inflammation Markers in Endothelial Cells

**Figure 3:**
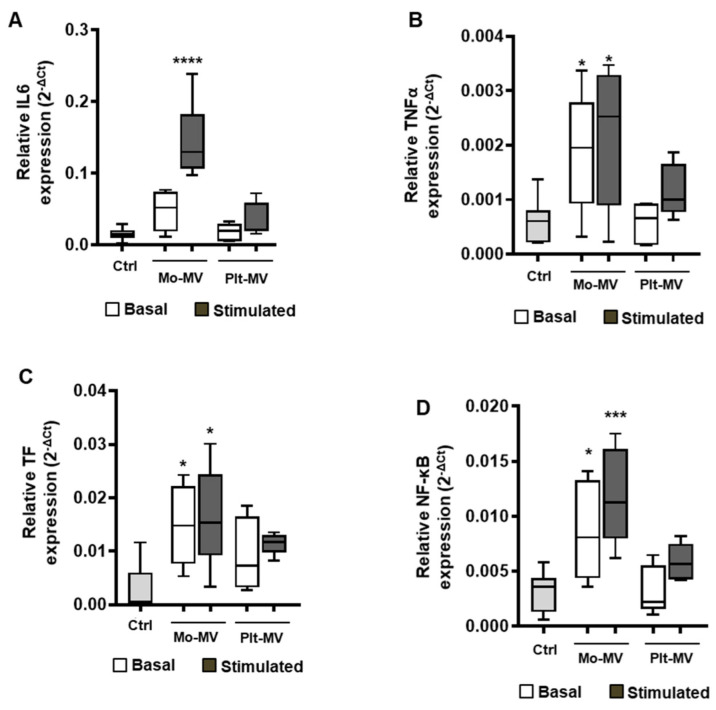
Mo- and Plt-MV effect on inflammation markers’ expression in human endothelial cells. hECV were stimulated for 6 h with MVs released from monocytes (*n* = 8) and platelets (*n* = 10) isolated from healthy subjects, under resting conditions (basal) or upon stimulation with LPS (10 μg/mL) or TRAP-6 (20 μM), respectively. Gene expression analysis for IL-6 (**A**), TNFα (**B**), TF (**C**), and NF-κB (**D**) was performed. Data are represented in box and whisker plot (min to max) and analyzed by one-way ANOVA with Dunnett’s test for multiple comparisons (TNFα), or Kruskall–Wallis with Dunn’s test for multiple comparisons (other genes). * *p* < 0.05; *** *p* < 0.001; **** *p* < 0.0001 vs. untreated cells (Ctrl).

We next assessed whether MVs induced a proinflammatory phenotype on endothelial cells in terms of IL-6, TNFα, TF, and NF-κB mRNA expression. Mo-MVs generated from resting or LPS-activated monocytes induced a significant increase of TNFα, NF-κB, and TF expression in hECVs (Figure 3B–D), whereas IL-6 was strongly increased by LPS-induced Mo-MVs only (Figure 3A). Conversely, MVs derived from unstimulated platelets did not influence the biosynthesis of both inflammation markers and Tissue Factor expression, whereas TRAP-6-induced Plt-MVs only lead to a slight increase in TNFα, NF-κB and IL-6 (Figure 3).

Overall, these data highlight the capacity of monocyte-derived MVs to regulate the endothelial inflammatory pathway, this occurring both with MV released from unstimulated or stimulated cells. In contrast, the endothelial inflammatory response is only slightly influenced by platelet-derived MVs.

### 2.4. Both Monocyte- and Platelet-Derived MVs Favor Monocyte Adhesiveness to Endothelial Cells

**Figure 4:**
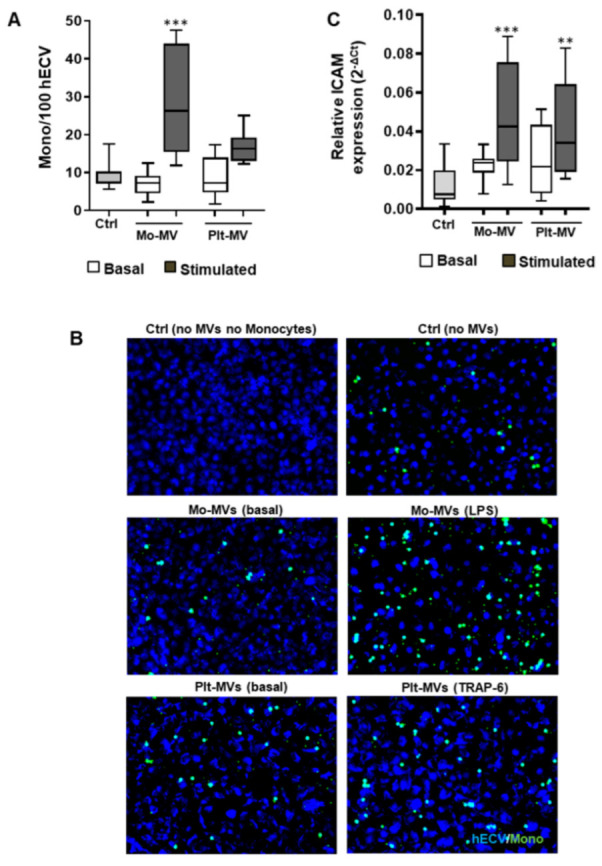
MV effects on monocytes adhesion to endothelial cells. hECV were stimulated for 6 h with MVs released from monocytes (Mo-MV) and platelets (Plt-MV) isolated from healthy subjects, at resting conditions (basal) or stimulated with LPS (10 μg/mL) or TRAP-6 (20 μM), respectively. (**A**) hECV cells were challenged 1 h with 200 MV/μL and then incubated 1 h 30 min with monocytes stained with calcein. Number of monocytes on hECV was quantified by ImageJ fluorescence. Data are represented in box and whisker plot (min to max) and are expressed as number of adherent monocytes on 100 hECV cells. (**B**) Representative images of adhesion assay: hECV nuclei stained with DAPI, monocytes stained by calcein, magnification 200×. (**C**) ICAM gene expression. Data are represented in box and whisker plot (min to max) of 2-ΔCt and analyzed by one-way ANOVA with Dunnett’s test for multiple comparisons. *n* = 8 experiments. ** *p* < 0.01; *** *p* < 0.001 vs. untreated control (Ctrl).

Evaluation of the effect of Plt- and Mo-MVs on the adhesive properties of endothelial cells showed that ICAM gene expression in hECV was not influenced by MVs generated from either type of unstimulated cells. It was instead significantly increased in LPS-induced Mo-MVs (Figure 4A), and this effect was paralleled by a significant increase (about threefold) in the number of adherent monocytes (Figure 4B,C). 

Similarly to what was observed in Mo-MVs, also hECV stimulation with Plt-derived MVs induced a significant upregulation of ICAM gene expression, with a resulting trend toward an increase of monocyte adhesion (Figure 4A–C).

### 2.5. The Amount of MVs Spontaneously Released by Cells from STEMI Patients Is Comparable to That of Stimulated Cells from Healthy Subjects

The above data suggest that an experimental pro-inflammatory environment triggers the production of MVs able to modulate endothelial function. Considering the well-known inflammatory background that characterizes patients with STEMI, we investigated, as a proof-of-concept, whether MVs released by cells isolated from STEMI patients (*n* = 7) recapitulated the effects observed with LPS- or TRAP-6-induced MVs from HS. 

Clinical and demographical characteristics of the enrolled patients are reported in Table 1. Specifically, all patients (60% female; mean age of 62 ± 15 years) were on antiplatelet therapy, and most of them had cardiovascular risk factors including hypertension, diabetes, or dyslipidemia.

The number of MVs released by STEMI resting monocytes and platelets were both significantly higher (235 ± 107 and 799 ± 363 MVs/µL, respectively) compared to the number of MVs produced by unstimulated cells from HS, being similar to the amount of MVs released after monocyte or platelet stimulation with LPS or TRAP-6 (291 ± 72 and 1115 ± 622 MVs/µL, respectively). Of note, the amount of MVs produced by STEMI-platelets was 3.5-fold higher compared to that released by patients’ monocytes (*p* = 0.008). 

### 2.6. STEMI Platelet-Derived MVs Induce Oxidative Stress and Inflammatory Phenotype on Endothelium While Monocyte-Derived MVs Affect Only Adhesion Properties

**Figure 5:**
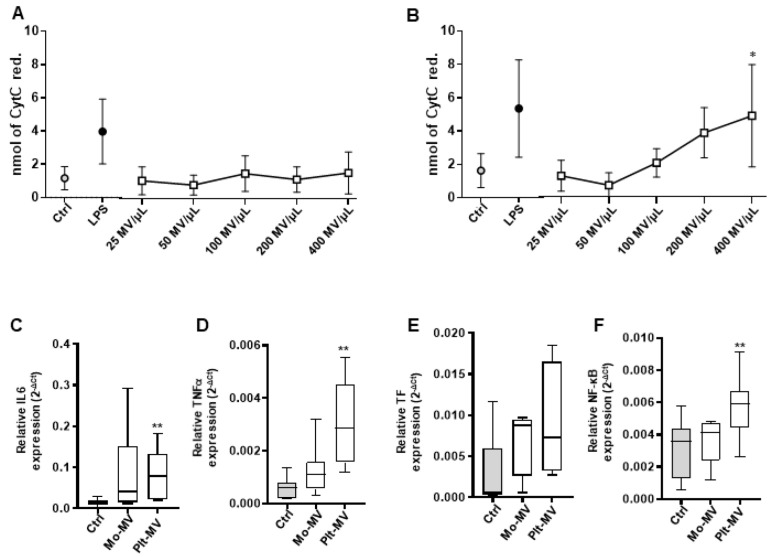
Effects of MVs released from monocytes and platelets isolated from STEMI patients on endothelial cells stress status. Superoxide anion production by hECV untreated (Ctrl), incubated overnight with LPS (as positive control), or incubated 30 min with the reported concentration of monocyte (Mo-) or platelet (Plt-)-derived MVs (**A**,**B**, respectively). Data are expressed as nmoles of cytochrome C reduced/10^6^ cells/30 min and are represented as mean ± SD of 5 independent experiments and analyzed by one-way ANOVA with Dunnett’s test for multiple comparison. * *p* < 0.05 vs. untreated cells (Ctrl). Gene expression of IL-6 (**C**), TNFα (**D**), TF (**E**), and NF-kB (**F**) in hECV stimulated 6 h with Mo- or Plt-MVs from STEMI patients. Data are represented in box and whisker plot (min to max) and analyzed by one-way ANOVA with Dunnett’s test for multiple comparison (TNFα, NF-κB) or Kruskall–Wallis with Dunn’s test for multiple comparison (IL-6). ** *p* < 0.01 vs. untreated cells (Ctrl).

We next characterized the effects of MVs spontaneously released by monocytes and platelets from STEMI patients on the endothelial behavior. Unlike what was expected, incubation of hECV with STEMI Mo-MVs did not induce an oxidative burst in vascular cells, even at the highest concentration used (Figure 5A). By contrast, MVs derived from platelets induced a significant concentration-dependent increase of superoxide anion production (Figure 5B), which was comparable to that triggered by MVs produced by stimulated HS cells.

In addition, unlike what was observed for HS-MVs, Plt-MVs but not Mo-MVs from STEMI patients induced a significantly increased expression of IL-6, TNFα, and NF-κB (Figure 5C–F) and a trend toward higher levels of TF mRNA expression in endothelial cells. 

**Figure 6:**
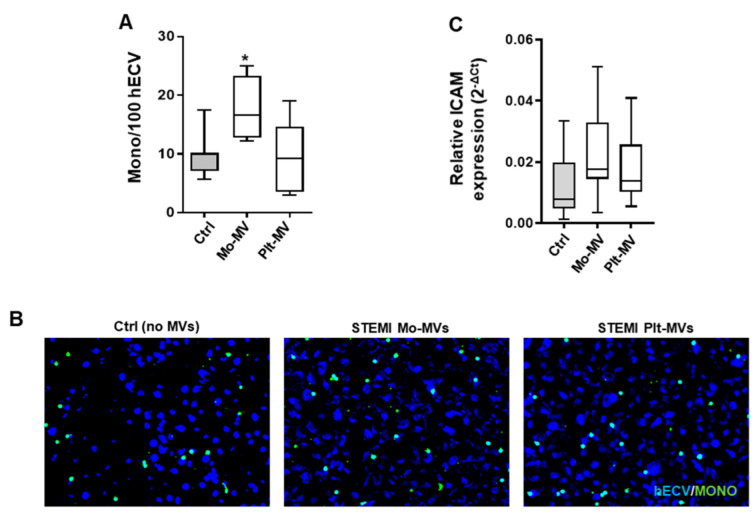
MV effects on monocytes adhesion to endothelial cells treated with MVs released by cells of STEMI patients. hECV were stimulated for 6 h with MVs released from monocytes (*n* = 5) and platelets (*n* = 7), isolated from STEMI patients. (**A**) hECV cells were challenged 1 h with 200 MV/μL and then incubated 1 h 30 min with monocytes (from healthy donors) stained with calcein. Number of monocytes on hECV was quantified by ImageJ fluorescence. Data are represented in box and whisker plot (min to max) and are expressed as number of adherent monocytes on 100 hECV cells. (**B**) Representative images of adhesion assay: hECV nuclei stained with DAPI, monocytes stained by calcein, magnification 200×. (**C**) ICAM gene expression. Data are represented in box and whisker plot (min to max) of 2-ΔCt. One way ANOVA with Dunnett’s test for multiple comparison was used to analyze data. * *p* < 0.05 vs. Ctrl (untreated cells).

Finally, only MVs derived from STEMI monocytes were able to double the number of adherent monocytes, and this was accompanied by a trend toward increased ICAM gene expression by endothelial cells (Figure 6).

Overall, our data suggest that MVs produced by platelets or monocytes from STEMI patients have distinct effects on the endothelial functions. In particular, MVs produced by STEMI platelets are able to influence processes as redox balance and inflammatory response of endothelial cells, whereas Mo-MVs act on the endothelium mainly by increasing its adhesive properties towards circulating monocytes. 

### 2.7. A Peculiar Surface Antigenic Profile Characterizes MVs Released from STEMI Cells

To circumstantiate the effects attributed to vesicles released by cells isolated from patients compared to those produced by in vitro stimulated cells, we assessed the MV antigenic signature by flow cytometry analysis.

**Figure 7:**
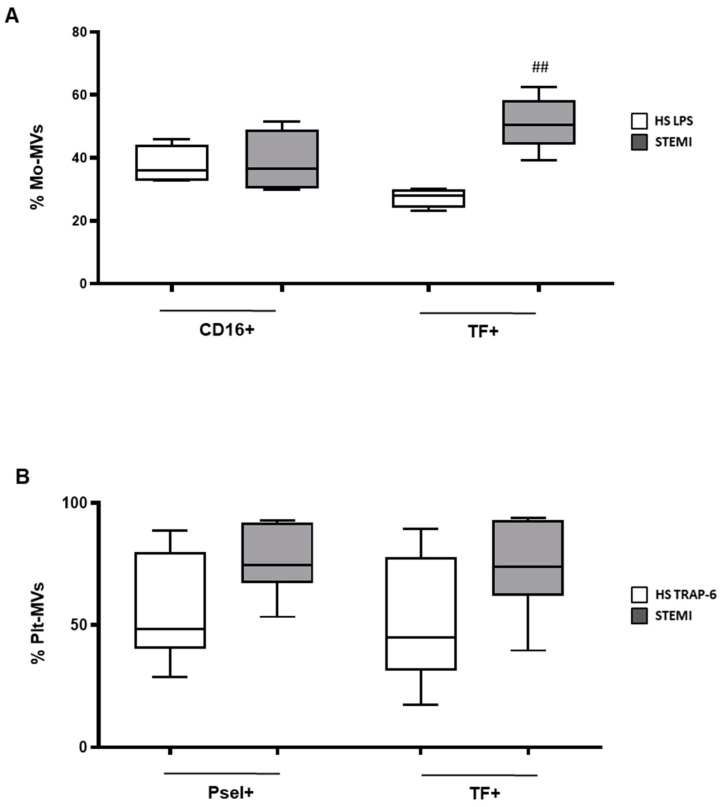
Surface markers expression in MVs released from stimulated monocytes (Mo; **A**) and platelets (Plt; **B**) of healthy subjects (HS, white box) and STEMI patients (grey box). Data are represented as box and whisker plot (min to max) of the percentage of MVs expressing the indicated markers and analyzed by Mann–Whitney U test. ## *p* < 0.05 vs. HS.

Data indicate that the percentage of CD16^+^ MVs released from STEMI monocytes was comparable to that from stimulated healthy cells (Figure 7A). However, levels of monocyte-derived TF^+^ MVs were significantly higher in STEMI compared to HS (Figure 7A), reflecting the increased prothrombotic phenotype of patients’ monocytes. Indeed, a double number of resting TF^+^ monocytes was found in STEMI patients compared to HS (3 ± 4.8% and 1.5 ± 1.1% of circulating monocytes, respectively *p* = 0.05). 

Of interest is also the evidence that unstimulated platelets of STEMI patients showed a trend toward higher production of Pselectin^+^ and TF^+^ MVs compared to HS (Figure 7B). The amount of procoagulant MVs produced by platelets reflected the increased number of circulating TF^+^ platelets detected in STEMI patients (5.4 ± 4.7 and 2.8 ± 1.4 TF^+^ platelets in STEMI and HS, respectively; *p* < 0.0001). 

Taken together, the data show that the expression of activation markers on MVs produced by cells from STEMI patients differs from that of HS cells even if both cell types have been exposed to a proinflammatory/prothrombotic milieu. In addition, they highlight the well described activated phenotype of platelets in acute coronary artery disease (CAD) [29], despite the ongoing antiplatelet therapy.

## 3. Discussion

This study explored for the first time the capacity of monocyte- and platelet-derived MVs prepared from the same donors—healthy subjects and patients with STEMI—to influence biological functions relevant in sustaining endothelial perturbation, i.e., redox balance, inflammation, and leukocyte adhesion. 

The data show that MVs derived from resting monocytes and platelets of healthy subjects do not induce endothelial perturbation, with the only exception of Mo-MVs that upregulate inflammatory proteins. 

MVs derived from HS monocytes and platelets upon in vitro activation, a condition that we used as surrogate of an in vivo inflammatory status, share the capacity to increase oxidative stress and the expression of adhesion proteins, whereas they behave differently in terms of upregulation of inflammatory proteins, function that is mainly exerted by monocyte-derived MVs. 

**Figure 8:**
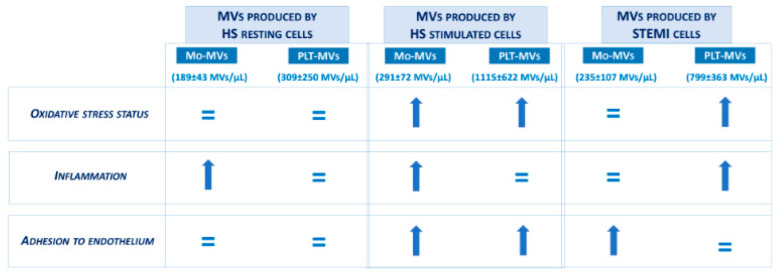
Summary of the endothelial effects exerted by MVs derived from healthy subject monocytes and platelets—under resting condition and upon stimulation—or spontaneously released by cells from STEMI patients.

Of interest, the capacity of MVs to modulate these endothelial functions was completely flipped when the effects of STEMI patients-derived MVs were tested. Indeed, upregulation of inflammatory proteins occurred only with platelet-derived MVs that were able to modify also the redox balance but not the adhesive properties. Basically, none of the endothelial functions studied were modulated by both STEMI monocyte- and platelet-derived MVs (Figure 8).

It is well known that inflammation has a central role in the pathogenesis of acute coronary syndromes (ACS) [30], and a strong correlation between elevated levels of inflammatory biomarkers and mortality in patients with ACS has been documented [31,32]. Monocytes are known to be cell mediators in response to cardiovascular injury [33]. They are a critical component of the inflammation response, producing high levels of cytokines, and are prompted to promote leukocyte-endothelium interactions which are among the main pathways affected by an inflammatory environment [33]. The regulation of leukocyte trafficking is mediated by ICAM-1 [34], which is highly induced by a variety of inflammatory cytokines [35]. Here, we provide evidence that MVs derived from LPS-treated monocytes increased ICAM-1 expression together with TNFα, IL-6, NF-κB, and TF upregulation, ultimately favoring monocyte adhesion. Interestingly, MVs spontaneously produced by STEMI-monocytes, while promoting leukocyte adhesion, did not induce the onset of an endothelial proinflammatory profile, thus highlighting differences between MVs released from pathologically primed and in vitro stimulated cells. This is not an unexpected result considering that monocytes and related MVs may play a role both in pro- and anti-inflammatory processes [36,37] and that this may be related to the stimulus (i) that has driven their release and composition [38,39]. 

As far as the anti-inflammatory role of monocyte is concerned, it has been suggested that leukocyte-derived MVs could actively participate, besides the initial onset of the inflammatory process, also in the resolution and tissue repair phases [40]. Recent evidence suggests indeed that circulating MVs from myocardial infarction patients are primed toward a cardioprotective potential upon TNFα-induced cell damage [41]. Keeping with this, Perretti and colleagues recently demonstrated that MVs produced by TNFα stimulated monocytes, while inducing endothelial ICAM-1 expression, did not upregulate IL-6 synthesis [42], unlike what observed in our experimental conditions with LPS treatment. 

Overall, the involvement of leukocyte-derived MVs in the resolution of vascular inflammation and cardiovascular diseases, although deserving further investigation, could justify why MVs of monocyte origin, unlike those derived from platelet, do not emerge as potential biomarkers for long term follow-up after a cardiovascular event [11,43]. 

Our data suggest that, in STEMI patients, studied within 48 h from the ischemic event, an endothelial inflammatory phenotype is sustained by platelet-derived MVs more than those derived from monocytes. In pathological conditions such as cardiovascular diseases, platelet-derived MVs, thanks to their capacity to transfer proteins, lipids, and nucleic acids to recipient cells, can actively participate in cell proliferation and migration in addition to the inflammatory response [44]. In particular, high P-selectin expression on MVs is associated to the degree of MV interaction with endothelial cells leading to the induction of oxidative burst in these cells [24,25,45,46]. Our results are in line with these observations showing that activated and STEMI derived-cells generate a greater number of P-selectin^+^ MVs; they also bring TF, thus potentially being able to promote thrombin formation [47] that, besides its central role in hemostasis and thrombosis, is involved in sustaining endothelial pro-inflammatory and pro-thrombotic phenotype [48]. Of note, while sharing a similar antigenic phenotype, platelet MVs from in vitro activated- and STEMI patient-derived cells, in agreement with what observed for monocyte-derived MVs, differently promote an increase of endothelial adhesive properties or cellular inflammation, respectively, while both promote the onset of a pro-oxidative burst.

The activated phenotype of platelet-derived MVs, which occurs in the enrolled STEMI despite the ongoing antiplatelet treatment, is particularly relevant from a clinical point of view as it may hold diagnostic and prognostic value. Indeed, studies from our group recently demonstrated that in CAD patients undergoing elective coronary artery bypass surgery, pre-operative levels of procoagulant, and activated platelet-derived MVs were predictive of bypass reocclusion in a 18-month follow-up [11]. Overall, these data, together with the evidence that a significant shift in the profile of circulating MVs is associated with STEMI prediction [49], support MVs as one of the causative factors of adverse cardiovascular outcomes including, possibly, coronary artery bypass reocclusion. 

Combined use of Aspirin and P2Y_12_ inhibitors is the standard treatment for reduction of high thrombotic risk in ACS patients [50]. However, while experimental studies showed that both P2Y_12_ inhibitors as well as aspirin, besides reducing platelet aggregation, are effective in preventing platelet-MV release [42,51] and in controlling the pro-inflammatory, oxidative stress, procoagulant, and adhesion properties of MVs [52], results from clinical trials are more limited and sometimes inconsistent [52,53,54,55,56]. It can be speculated that mechanisms escaping those controlled by aspirin and P2Y_12_ inhibitors may account for the variability of in vivo results as well as for the effects mediated by STEMI-derived MVs we observed in the present study. Alternatively, as it has been demonstrated that the inhibitory effects of clopidogrel on platelet MV release were related to the clopidogrel active metabolite plasma concentration [53], inadequate effectiveness of drug therapy may be implicated in the MV effects in STEMI. However, although we have not assessed in our study the residual platelet reactivity, this hypothesis is weakened by the consistency of the data obtained from the enrolled patients, and this makes a sub-optimal pharmacological response unlikely.

## 4. Study Limitations

Our findings should be interpreted in the context of their limitations. First, the relatively low number of patients enrolled in this study makes the results presented a proof-of-concept that will have to be confirmed by studies enrolling a larger number of patients. Second, in the in vitro production of MVs by activated monocytes and platelets, only one agonist and only one stimulation time were used for each cell type. We cannot exclude therefore that MVs produced by using different experimental settings—in terms of agonists and stimulation time—would have modified endothelial function differently considering, as previously mentioned, the knowledge that the same cell may produce different MVs with different functions in relation to the stimuli used. Similarly, the timing of the sample collection with respect to the onset of the acute event could influence the pattern and the effects of released MVs. Thus, MVs produced by STEMI cells sampled earlier or later compared to the timing used in this study would provide important additional information useful to better understand the role of MVs on endothelial functions in this clinical setting. Furthermore, they may also help explain the discrepancy between in vitro and in vivo data.

## 5. Materials and Methods

### 5.1. Patients and Healthy Subjects’ Enrollment

Fifteen healthy subjects (HS) were recruited among the hospital staff (mean age 36 ± 7; 26.7% male). All the enrolled HS had no clinical evidence of inflammatory, neoplastic or cardiovascular disease and did not take antiplatelet drugs within 10 days before blood donation.

Seven patients affected by ST segment elevation myocardial infarction (STEMI) were enrolled for comparison at Centro Cardiologico Monzino in Milan, Italy. STEMI was defined as chest pain at rest with documented persistent ST-segment elevation with enzymatic evidence of myocardial necrosis. The last spontaneous episode of chest pain had to have occurred within the 48 h for admission into the study. The exclusion criteria were: age > 80 years, valvular heart disease, atrial fibrillation, thyrotoxicosis, a history of hemorrhagic diathesis, platelet disorder or thrombocytopenia, malignancies, inflammatory diseases, major surgery or trauma within the preceding month, or severe liver disease.

All the subjects gave their informed consent to participate in the study. The investigation conforms to the principles outlined in the Declaration of Helsinki and was approved by the Ethical Committee at Centro Cardiologico Monzino (NCT00755248).

### 5.2. Cells Isolation

Blood was collected by venipuncture from healthy subjects and STEMI patients in tubes containing sodium citrate. Specimens were centrifuged for 10 min at 100 g, and the platelet-rich plasma (PRP) was transferred into a new tube. For platelets isolation, the PRP was diluted in Tyrode’s buffer (134 mM NaCl, 12 mM NaHCO_3_, 2.9 mM KCl, 0.34 mM Na_2_HPO_4_, 1 mM MgCl_2_, 10 mM HEPES, pH 7.4) 0.25% BSA (Sigma-Aldrich, St. Louis, Missouri, USA) and washed twice. Then, the platelets were resuspended in Tyrode’s buffer 0.25% BSA and 2.5 mM CaCl_2_ and stimulated for MVs production. For monocyte isolation, the blood depleted of PRP was reconstituted with RPMI 1640 (Sigma-Aldrich, St. Louis, Missouri, USA) 0.38% sodium citrate and stratified by ficoll (GE Healthcare, Chicago, Illinois, USA) gradient. Then, the cells were recovered by thin suction at the interface. Purified monocyte populations were obtained by adhesion (90 min, 37 °C, 5% CO_2_) in serum free RPMI 1640 medium supplemented with 2 mM glutamine, 1% HEPES and 1% antibiotics. 

### 5.3. Microvesicles Production 

Microvesicles from platelets (Plt-MVs; 3 × 10^8^) have been generated after 60 min cell incubation at 37 °C with or without TRAP-6 20 µM. Monocytes (Mo; 1 × 10^6^) MVs were obtained after overnight incubation of cells at 37 °C without or with LPS 10 µg/µL. MVs were then recovered from culture medium by a 20 min centrifugation at 20,000× *g*.

### 5.4. Flow Cytometry

MV characterization was performed by flow cytometry as previously described [11] with minor changes. Briefly, fifty microliters of each sample were diluted in 0.22 µm-pore-size-membrane-filtered buffer containing Hepes (10 mM), NaCl (140 mM), and CaCl_2_ (2.5 mM) at pH 7.4 (Annexin Binding Buffer, ABB) with PPACK (15 µM) to prevent clot formation. To identify intact MVs, samples were incubated with calcein AM (10 µM; Invitrogen, Waltham, Massachusetts, USA) at 37 °C in the dark for 25 min. Saturating concentrations of MoAbs were then added for 15 min at room temperature in the dark. Antibodies were combined in different mixes as follows: mix 1, containing αCD41, αTF moAbs; mix 2, containing αCD41 and αP-selectin moAbs; and mix 3, containing αCD14, αCD16 and αTF moAbs. Samples were immediately analyzed on Gallios flow cytometer (Beckman Coulter, Brea, CA, USA) equipped with four solid-state lasers at 488, 638, 405, and 561 nm, with an enhanced wide forward angle light scatter optimized for MV detection. Flow-check Pro Fluorospheres (Beckman Coulter, Brea, CA, USA) were daily used according to the manufacturer’s instruction to monitor cytometer performance. Megamix-FSC Plus beads (0.5, 0.9, 3 µm; Biocytex, Marseille, France) were used to define the analysis gate and BD Trucount tubes^TM^ (Becton Dickinson, Franklin Lakes, NJ, USA) to have the absolute count of MVs. All samples were processed by the same experienced operator.

### 5.5. Cell Culture

Human vascular endothelial (hECV) cells [57] were routinely cultured with DMEM/F12 (Sigma-Aldrich, St. Louis, Missouri, USA) added by 10% fetal bovine serum (FBS), 1% glutamine and 1% penicillin-streptomycin (all Invitrogen, Waltham, MA, USA). 

### 5.6. Quantitative Real-Time PCR

For real-time PCR, hECV cells were incubated 6 h at 37 °C with 200 MV/μL. Total RNA was isolated by Tri-Reagent and the amount and purity were quantified at the spectrophotometer (Nanodrop, Thermo Fisher, Waltham, Massachusetts, USA) by measuring the optical density at 260 and 280 nm. cDNA synthesis was performed using a high-capacity cDNA re-verse transcription kit (Applied Biosystems, Waltham, Massachusetts, USA) according to the manufacturer’s instructions. For gene expression analysis, SensiFast No-ROX kit (Bioline, Memphis, Tennessee, USA) was used. The primers used were: IL-6 forward 5′-GGAGACTTGCCTGGTGAAAA-3′ and reverse 5′- GTCAGGGGTGGTTATTGCAT-3′, NF-κB forward 5′-ACACCGTGTAAACCAAAGCC-3′ and reverse 5′-CAGCCAGTGTTGTGATTGCT-3′, TNFα forward 5′-CATGATCCGGGACGTGGAGC-3′and reverse 5′-CTGATTAGAGAGAGGTCCCTG-3′, TF forward 5′-TGATGTGGATAAAGGAGAAAACTACTGT-3′ and reverse 5′-TCTACCGGGCTGTCTGTACTCTT-3′, ICAM forward 5′-GGCTGGAGCTGTTTGAGAAC-3′ and reverse 5′-ACTGTGGGGTTCAACCTCTG-3′ and GAPDH forward 5′-AACGTGTCAGTGGTGGACCTG-3′ and reverse 5’-AGTGGGTGTCGCTGTTGAAGT-3′.

### 5.7. Superoxide Anion (O^2−^) Production

hECVs were treated for 30 min with several concentrations of MV (25, 50, 100, 200, and 400 MVs/μL). Cells stimulated with LPS 1 μM were used as positive control. Superoxide anion production was then evaluated by the superoxide dismutase (SOD)-sensitive cytochrome C reduction assay, and results are expressed as nmoles cytochrome C reduced/10^6^ cells/30 min, using an extinction coefficient of 21.1 mM as previously described [58]. 

### 5.8. Adhesion Assay

100,000 hECV cells were stimulated with 200 MVs/µL for 1 h at 37 °C. Concurrently, cells were incubated with DAPI (Sigma-Aldrich, St. Louis, MO, USA) for nuclear staining. Then, MVs were removed, and 250,000 calcein stained human primary monocytes were seeded on stimulated cells for 90 min. After incubation, not adherent monocytes were removed by PBS 1× washing and images acquired at the EVOS FLoid Cell Imaging Station microscope (Invitrogen, Waltham, MA, USA).

### 5.9. Statistical Analysis

All samples were first tested for normality (Shapiro–Wilk test) and for homogeneity of variance (Levene’s test). When possible, statistical significance was assessed by parametric tests, i.e., linear regression and t test or one-way analysis of variance (ANOVA) (followed by Tukey’s or Dunnett’s post hoc test) in the case of continuous and categorical independent variables, respectively. Otherwise, nonparametric alternatives were used, as detailed in the figure legends.

## 6. Conclusions

In conclusion, results presented in this study underline the different impact on endothelial functions of MVs derived from activated monocytes and platelets compared to those produced by resting cells, the latter not affecting endothelial resilience except for Mo-MVs that upregulate inflammatory proteins. This effect is not observed with MVs produced by STEMI cells, underlining that in vitro stimulation does not always mirror the pathological condition.

Finally, our data further strengthen platelet-derived MVs, more than those derived from monocytes, as one of the potential actors of impaired endothelial function in the acute phase of STEMI.

## Figures and Tables

**Table 1:** Clinical and demographic characteristics of the enrolled STEMI patients.

	STEMI Patients (*n* = 7)
Men, *n* (%)	3 (43)
Age, years	63 ± 13
Hypertension, *n* (%)	6 (86)
Diabetes, *n* (%)	3 (43)
Dyslipidemia, *n* (%)	5 (71)
Pharmacological therapy	
Aspirin, *n* (%)	7 (100)
P2Y12 inhibitors, *n* (%)	7 (100)
Calcium antagonists, *n* (%)	2 (29)
ACE inhibitors, *n* (%)	4 (57)
Statins, *n* (%)	5 (71)

## Data Availability

The data presented in this study are available on request from the corresponding author.

## Abbreviations

ACS: acute coronary syndrome; CAD: coronary artery disease; hECV: human vascular endothelial cells; HS: heathy subjects; ICAM-1: intercellular adhesion molecule-1; IL-6: interleukin-6; LPS: lipopolysaccharides; MVs: microvesicles; NF-kB: nuclear factor kappa-light-chain-enhancer of activated B cells; STEMI: ST-elevation myocardial infarction; TF: tissue factor; TNFα: tumor necrosis factor alpha; TRAP-6: thrombin receptor activator peptide 6.
